# Polymicrobial Oral Infection with Four Periodontal Bacteria Orchestrates a Distinct Inflammatory Response and Atherosclerosis in ApoE^null^ Mice

**DOI:** 10.1371/journal.pone.0143291

**Published:** 2015-11-30

**Authors:** Sasanka S. Chukkapalli, Irina M. Velsko, Mercedes F. Rivera-Kweh, Donghang Zheng, Alexandra R. Lucas, Lakshmyya Kesavalu

**Affiliations:** 1 Department of Periodontology, College of Dentistry, University of Florida, Gainesville, Florida, United States of America; 2 Department of Oral Biology, College of Dentistry, University of Florida, Gainesville, Florida, United States of America; 3 Division of Cardiovascular Medicine, Department of Medicine, Gainesville, Florida, United States of America; 4 Department of Molecular Genetics & Microbiology, College of Medicine, University of Florida, Gainesville, Florida, United States of America; Boston University, UNITED STATES

## Abstract

Periodontal disease (PD) develops from a synergy of complex subgingival oral microbiome, and is linked to systemic inflammatory atherosclerotic vascular disease (ASVD). To investigate how a polybacterial microbiome infection influences atherosclerotic plaque progression, we infected the oral cavity of ApoE^null^ mice with a polybacterial consortium of 4 well-characterized periodontal pathogens, *Porphyromonas gingivalis*, *Treponema denticola*, *Tannerealla forsythia* and *Fusobacterium nucleatum*, that have been identified in human atherosclerotic plaque by DNA screening. We assessed periodontal disease characteristics, hematogenous dissemination of bacteria, peripheral T cell response, serum inflammatory cytokines, atherosclerosis risk factors, atherosclerotic plaque development, and alteration of aortic gene expression. Polybacterial infections have established gingival colonization in ApoE^null^ hyperlipidemic mice and displayed invasive characteristics with hematogenous dissemination into cardiovascular tissues such as the heart and aorta. Polybacterial infection induced significantly higher levels of serum risk factors oxidized LDL (p < 0.05), nitric oxide (p < 0.01), altered lipid profiles (cholesterol, triglycerides, Chylomicrons, VLDL) (p < 0.05) as well as accelerated aortic plaque formation in ApoE^null^ mice (p < 0.05). Periodontal microbiome infection is associated with significant decreases in *Apoa1*, *Apob*, *Birc3*, *Fga*, *FgB* genes that are associated with atherosclerosis. Periodontal infection for 12 weeks had modified levels of inflammatory molecules, with decreased Fas ligand, IL-13, SDF-1 and increased chemokine RANTES. In contrast, 24 weeks of infection induced new changes in other inflammatory molecules with reduced KC, MCSF, enhancing GM-CSF, IFNγ, IL-1β, IL-13, IL-4, IL-13, lymphotactin, RANTES, and also an increase in select inflammatory molecules. This study demonstrates unique differences in the host immune response to a polybacterial periodontal infection with atherosclerotic lesion progression in a mouse model.

## Introduction

Chronic periodontal disease (PD) is an inflammatory disorder in which several subgingival microbes and their virulence factors synergistically alter host cytokine and chemokine responses. Subgingival dental plaque is juxtaposed upon host periodontal tissue, and serves as a primary reservoir of a microbial consortium, thereby providing a constant challenge to the host immune system. Disease progression however, is dependent on a dysbiotic oral chronic bacterial infection that can enhance host inflammatory processes with destruction of the supporting structures of teeth, including the gingival epithelium, periodontal ligament, connective tissue, and alveolar bone [1]. The periodontal tissues use two completely different strategies to contend with the constant presence of microbial stimulation.

Factors driving microbial dysbiosis are not yet clear, but may be due to actions of a central or keystone pathogen, a single microbial species, such as *P*. *gingivalis* [2–3]. However, disease pathology is due to shifts in microbial composition and increased microbial burden, rather than to the direct actions of a single species [2]. One mechanism by which the commensal oral microbiome may disrupt periodontal tissue homeostasis is by capitalizing on the immunomodulatory phenotype of each of the major periodontal bacteria, specifically *P*. *gingivalis*, *T*. *denticola*, and *T*. *forsythia*. These immunomodulatory functions are designed to work against the host innate response [4]. Additionally, it is well established that periodontal disease has a polymicrobial etiology, a focus of synergistic polybacterial infection that can induce disease. These polymocrobial periodontal infections have been neglected due to a lack of understanding of this complex disease and due to prior focus of research studies on *in vivo* infection with single bacterial species (the mono-infection theory). There are in fact over 400 commensal organisms in the oral cavity and thus polymicrobial infection is a more natural process with potential for causing disease progression, whether oral or cardiovascular.

A number of studies have reported differences in pathological outcomes of mixed or multispecies infections compared to monospecies infection in the oral cavity [5–7]. Mixed infection with *P*. *gingivalis* and *T*. *denticola* induces greater alveolar bone resorption and apical junctional epithelial migration in a rat oral infection model when compared to either *P*. *gingivalis* or *T*. *denticola* infection alone [6], while *T*. *forsythia* and *F*. *nucleatum* mixed oral infection in mice induced greater leukocyte infiltration of gingival tissues, osteoclast activation and alveolar bone resorption than *T*. *forsythia* or *F*. *nucleatum* alone [7]. Transcriptional profiling of calvarial bone and soft tissue in response to polybacterial infection [8] reveals differences in gene expression in bone and soft tissue compared to monoinfection with *P*. *gingivalis* [9], *T*. *denticola* [10], and *T*. *forsythia* [11]. These studies suggest that host immune and physiologic synergistic interaction to a polybacterial infection is distinct from individual infections.

Our recent investigations in to the ability of a polybacterial consortium of periodontal infection with three major bacteria in ApoE^-/-^ mice has led to the detection of a positive correlation between the number of bacterial species detected in mouse aortas and total area of atherosclerotic plaque [12], suggesting that atherosclerosis is a multifactorial polymicrobial infection-driven disease [13]. In support of this, genomic DNA of numerous oral and gut microorganisms have been detected in atherosclerotic plaques [13–15], indicating polygenic or polymicrobial infections which may drive inflammation and plaque progression. Additionally, it was recently reported that bacteria in human atherosclerotic carotid arteries exist as polymicrobial biofilms [16], further demonstrating the need to investigate *in vivo* how polybacterial consortiums modulate vascular pathology.

In this study, we explore the potential mechanisms by which the dysbiosis mediated by oral polybacterial infection with periodontal pathogens *P*. *gingivalis*, *T*. *denticola*, *T*. *forsythia* and the “bridging bacterium” *F*. *nucleatum* initiates periodontal disease spreads through the vasculature, elevates systemic inflammatory burden, and promotes atherosclerosis in a hyperlipidemic ApoE^-/-^ mouse. We have detected several key molecules involved in the inflammatory cascade contributing to atherosclerosis development in the context of polybacterial infections. The current study further expands our knowledge in the field of polybacterial-mediated PD and the role it plays in modulation of systemic physiology and development of atherosclerosis.

## Materials and Methods

### Microbial Strains and bacterial Inoculum

*P*. *gingivalis* ATCC 53977, *T*. *denticola* ATCC 35404, *T*. *forsythia* ATCC 43037 and *F*. *nucleatum* 49256 were routinely cultured anaerobically at 37°C as described previously [12, 17]. Bacterial concentration was determined by OD_600_ and cells were resuspended in equal proportions in reduced transport fluid (RTF) at 10^10^ cells/mL [12]. To prepare the bacterial suspension for infection, *P*. *gingivalis* was mixed with an equal quantity of *T*. *denticola* for 5 min; subsequently, *T*. *forsythia* and *F*. *nucleatum* were added to the culture tube containing *P*. *gingivalis* and *T*. *denticola*, and cells were mixed thoroughly and allowed to interact for an additional 5 min. The polybacterial mixture was then mixed with an equal volume of 8% (w/v) sterile carboxymethylcellulose (CMC; Sigma-Aldrich, St. Louis, MO) in phosphate buffered saline (PBS), and this mixture was used for periodontal infection (2.5 x 10^9^ bacteria *per* mL) in ApoE^-/-^ mice as described previously [12,17].

### Mouse Strain and periodontal Infection

Eight-week old male ApoE^-/-^ B6.129P2-*Apoe* ^*tm1Unc*^/J mice were obtained from Jackson Laboratories (Bar Harbor, ME) and were acclimated as described [12]. Twenty-four mice were randomly assigned to the polybacterial infection group, and 24 mice to the sham-infection group. Mice were orally infected by oral lavage with 10^9^ total cells in 8% CMC in RTF as previously described [12]. Sham-infected mice were mock-infected with 8% CMC in RTF only. Sera, jaws and internal organs were collected following euthanasia at 12 and 24 weeks. All mice procedures were conducted in accordance with the guidelines of the University of Florida Institutional Animal Care and Use Committee (IACUC, protocol #201304539). The University of Florida has an Assurance with OLAW (Office of Laboratory Animal Welfare) and follows United States PHS (Public Health Services) policy, the Animal Welfare Act and Animal Welfare Regulations, and the Guide for the Care and Use of Laboratory Animals. The University of Florida is also AAALAC (Association for Assessment and Accreditation of Laboratory Animal Care) accredited.

### Gingival Plaque Sampling and genomic DNA Detection

Mouse gingival plaque samples were collected using sterile cotton swabs. One pre-infection sample was collected, and eight post-infection plaque samples were collected four days after each infection cycle from all the infected mice. Subsequently, PCR was performed using the following 16s rDNA gene bacterial species-specific primers (*P*. *gingivalis*): 5`-TGTAGATGACTGATGGTGAAAACC-3`(forward), 5`-ACGTCATCCCCACCTTCCTC-3’(reverse); (*T*.*denticola)* TAATACCGAATGTGCTCATTTACAT-3`(forward), 5`-CTGCCATATCTCTATGTCATTGCTCTT-3`(reverse); (*T*. *forsythia*) 5`-AAAACAGGGGTTCCGCATGG-3`(forward), 5`-TTCACCGCGGACTTAACAGC-3`(reverse), and (*F*. *nucleatum*) 5’-TAAAGCGCGTCTAGGTGGTT-3’ (forward), 5’-AC**A**GCTTT**G**CGACTCTCTGT-3’ (reverse) [17]. Bold letters were altered from the reference for a 100% match with strain ATCC 49256. Cycling was performed with a Bio-Rad thermal cycler as described [12].

### Detection of Bacterial Genomic DNA in Internal Organs

Heart, aorta, liver, spleen, kidney, and lung tissues were collected after euthanasia in Reduced transport fluid (RTF) and were stored at -80°C till further use. For analysis of bacterial genomic DNA, tissues were thawed, homogenized using a mechanical tissue disruptor (QIAGEN TissueRuptor^®^, Valencia, CA) and genomic DNA extracted using QIAGEN DNEasy blood and tissue kit (QIAGEN, Valencia, CA) as per the protocol described in the kit. Polymerase chain reaction (PCR) was performed using the same primers and cycling procedure described above [12].

### Serum IgG and IgM Antibody Analysis

Blood was collected by cardiac puncture for each mouse under anesthesia when euthanized and sera was separated and used to determine IgG and IgM antibody concentrations, using a standard ELISA protocol [5–6, 12, 17]. Mean antibody titer values of infected mice were divided by mean antibody titer values of sham-infected mice, and the quotient represents the fold change in mean specific antibody titer produced by polybacterial infection. Graphs demonstrate mean fold-change in specific antibody titer of infected mice. Statistically significant differences between mean specific-antibody titers of infected and sham-infected groups were determined by unpaired two-tailed Student’s t test, and are indicated above fold-change values on the graph.

### Morphometric Analysis of Alveolar Bone Resorption (ABR)

Histomorphometric analysis of horizontal ABR was performed as previously described [12, 18–19], with the following modifications. Cementum was not stained with methylene blue prior to measuring alveolar bone resorption. Total ABR was calculated by adding alveolar bone resorption calculated on maxilla palatal, maxilla buccal, and mandible lingual alveolar bone. Intrabony defect was measured as either present or absent on the palatal and buccal sides of all three molar teeth, and the % of defect was determined by dividing the number of intrabony defects by the total number of sites measured. Measurements represent the average bone defects determined by three independent viewers blinded to the groups [19].

### Morphometric Analysis of Aortic Atherosclerosis

The heart, aortic arch, thoracic aorta, and abdominal aorta were harvested from infected (n = 12 hearts, 6 aortas) and control mice (n = 12 hearts, 6 aortas). Two transverse 5 μm sections from each of three aortic regions (aortic valve with ascending aorta, as well as thoracic, and abdominal aorta) were cut on a Leica EG 1160 Cryostat (Leica Microsystems Inc, Bannockburn, IL) for Hematoxylin-eosin-staining and histological morphometric analysis. Plaque area was measured using an Olympus DP7 color video camera attached to an Olympus BX51 microscope and the Image Pro MC 6.0 software program (Olympus America, Center Valley, PA). The mean plaque area, lumen, and internal elastic lamina (IEL) areas, intimal and medial thickness ratios, as well as cell counts were measured by morphometric analysis using the Image Pro system MC 6.0 software program (Olympus America, Center Valley, PA,USA) by a reviewer blinded to the oral infection groups as previously described [19].

### Immunohistochemical Analysis of Inflammatory Cell Infiltration

In order to detect infiltration of the aortic tissue (n = 6 infected and sham-infected) with CD3^+^ T cells immunohistochemistry was performed as previously described [19]. The number of positively stained cells per high power field (HPF:100X) were counted by a reviewer blinded to the groups with cell counts performed in three separate areas for each stained section and the mean cell count per HPF calculated for each mouse artery.

### Fluorescent *in situ* Hybridization (FISH)

FISH was performed on formalin-fixed paraffin-embedded aortic, kidney, liver and spleen tissue sections of infected (n = 6) and sham-infected (n = 6) mice using Alexafluor-568 (Invitrogen, Carlsbad, CA) labeled 16s rRNA bacterial species-specific oligonucleotide probes, POGI (Sunde *et al*., 2003) 5’-CAATACTCGTATCGCCCGTTATTC-3’, TDEN 5’- CATGACTACCGTCATCAAAGAAGC -3’, B(T)AFO 5’-CGTATCTCATTTTATTCCCCTGTA-3’, and FUSO 5’-CTAATGGGACGCAAAGCTCTC-3’, which detect *P*. *gingivalis*, *T*. *denticola*, *T*. *forsythia*, and *F*. *nucleatum*, respectively. FISH was performed as previously described [12, 18–19].

### Recovery of Viable Bacteria from Heart and Aorta

Upon sacrifice a small section of heart (n = 6) and aorta (n = 3) from mice in both groups at both 12 and 24 weeks were homogenized and plated on blood agar, or incubated with THP-1 cells for 3 hours, then plated on fastidious anaerobe agar (FAA) [20]. Both blood agar and FAA plates were incubated for 14 days in an anaerobic chamber, and were monitored daily for growth.

### Serum Atherosclerosis Risk-factor Measurements

Assessment of the serum lipid profile [total cholesterol, total triglycerides, chylomicrons, very low density lipoprotein (VLDL), low density lipoprotein (LDL), high density lipoprotein (HDL), oxidized LDL (oxyLDL), serum amyloid A (SAA), and nitric oxide (NO) was performed on sera collected on sacrifice from polybacterial-infected (n = 6) and sham-infected (n = 6) mice. Thirty microliters of serum from 24 week-infected (n = 6) and sham-infected (n = 6) mice was sent for cholesterol and triglyceride analysis by Skylight Biotech Inc (Akita, Japan). Analysis of oxyLDL, SAA and NO was performed as previously described [18].

### Mouse Atherosclerosis RT^2^ Profiler PCR array

Expression levels of 84 pre-selected atherosclerosis-related genes were examined in aortas of infected (n = 3) and sham-infected (n = 3) mice by qRT-PCR with the RT^2^ Profiler Mouse Atherosclerosis PCR Array (SABiosciences, Valencia, CA). Samples were processed and analyzed as previously described [19].

### Evaluation of Inflammatory Cytokines in Mouse Serum

Mouse sera from 12 and 24 week infected (n = 10) and sham-infected (n = 10) mice were pooled and used to detect 40 different cytokines on the Ray Biotech Mouse Inflammatory Cytokine Array (RayBiotech, Inc, Norcross, GA), as previously described [18].

### Flow Cytometry

At 12 week sacrifice, whole spleens of 6 infected and 6 sham-infected mice were collected in RPMI-1640 supplemented with 10% FBS, 1% sodium pyruvate, 1% non-essential amino acids and 1% L-glutamine (RPMI-complete). Spleens were homogenized and cells frozen in RPMI-10% DMSO at -80°C for analysis. For flow cytometric analysis of T cell subtypes, frozen samples were thawed in a 37°C water bath, cell counts normalized to10^5^ in FACS buffer (PBS-1% FBS-0.1% EDTA) in wells of a FACS plate (Thermo Scientific, Waltham, MA). The antibodies were added to wells at 1:100 and incubated for 30 min at 4°C. The antibodies detected the following targets: CD3-BrilliantViolet 421 (Biolegend 100227) to mark CD3^+^ T cells, CD4-PE-Cy7 (eBioscience 25–0041) to mark CD4^+^ T cells, IFNγRα-PE (eBioscience 12–1191) to mark Th1 cells, IL-4Rα-Fluorescein (R&D Systems FAB503F) to mark Th2 cells, and IL-17Rα-APC (eBioscience 17–7182) to mark Th17 cells. After incubation, cells were washed in FACS buffer, and fixed in 2% paraformaldehyde. Samples were detected on LSRII Flow Cytometer (BD Biosciences) using FACSDiva software (BD Biosciences), and analysis performed using FCSExpress 4.0 software (DeNovo).

### Statistical Analysis

The statistical analysis of ELISA, serum lipid profile, SAA, NO, flow cytometry, and horizontal alveolar bone resorption were performed using a unpaired, two-tailed Student’s T test with GraphPad Prism software v. 5, and *P* < 0.05 were considered statistically significant. ELISA, flow cytometry, and horizontal ABR graphs show mean with standard deviation. Aortic histology measurements and immunohistochemistry cell counts were analyzed by ANOVA with the Statview program and *post hoc* PLSD analysis, and graphs and data represented as mean with standard error.

## Results

### Gingival Infection induces natural pattern of bacterial colonization, antibody responses and increased ABR

Mice were infected with polybacterial inoculum, following which the gingival surface was swabbed four days after each infection cycle and species-specific PCR was performed on these samples to test for the presence or absence of bacterial genomic DNA to monitor bacterial colonization. Following the second infection cycle, a natural pattern of bacterial colonization was observed, which was initially dominated by the early colonizer *F*. *nucleatum*, followed by late colonizers *P*. *gingivalis*, *T*. *denticola* and *T*. *forsythia* (Table 1). Although swabbing of the gingival surface may not be reflective of the subgingival microflora, the presence of these anaerobic species 4 days after inoculation suggests that the organisms were viable and adherent, as dead or non-adherent organisms would be removed by eating, drinking and saliva.

**Table 1:** Distribution of ApoE^-/-^ mice gingival plaque samples positive for *P*. *gingivalis*, *T*. *denticola*, *T*. *forsythia*, *F*. *nucleatum* genomic DNA by PCR.

Group	Positive gingival plaque samples				
	12 week infection (n = 28)				24 week infection (n = 16)
	1^a^	2	3	4	5
*Pg/Td/Tf/Fn*	0/0/0/0	16/16/16/19	1/17/10/2	N/D	0/10/12/14
Sham-infection	0/0/0/0	N/D	N/D	N/D	0/0/0/0

^a^ Indicate number of infections at which gingival plaque samples were collected to determine bacterial colonization by PCR.

Polybacterial infection cycles 1–4 had n = 28 mice and infection cycles 5–8 had n = 16 mice. N/D–samples were not collected. Samples were not collected following the 6^th^-8^th^ infections to allow the bacterial biofilm to develop without disruption.

**Fig 1:**
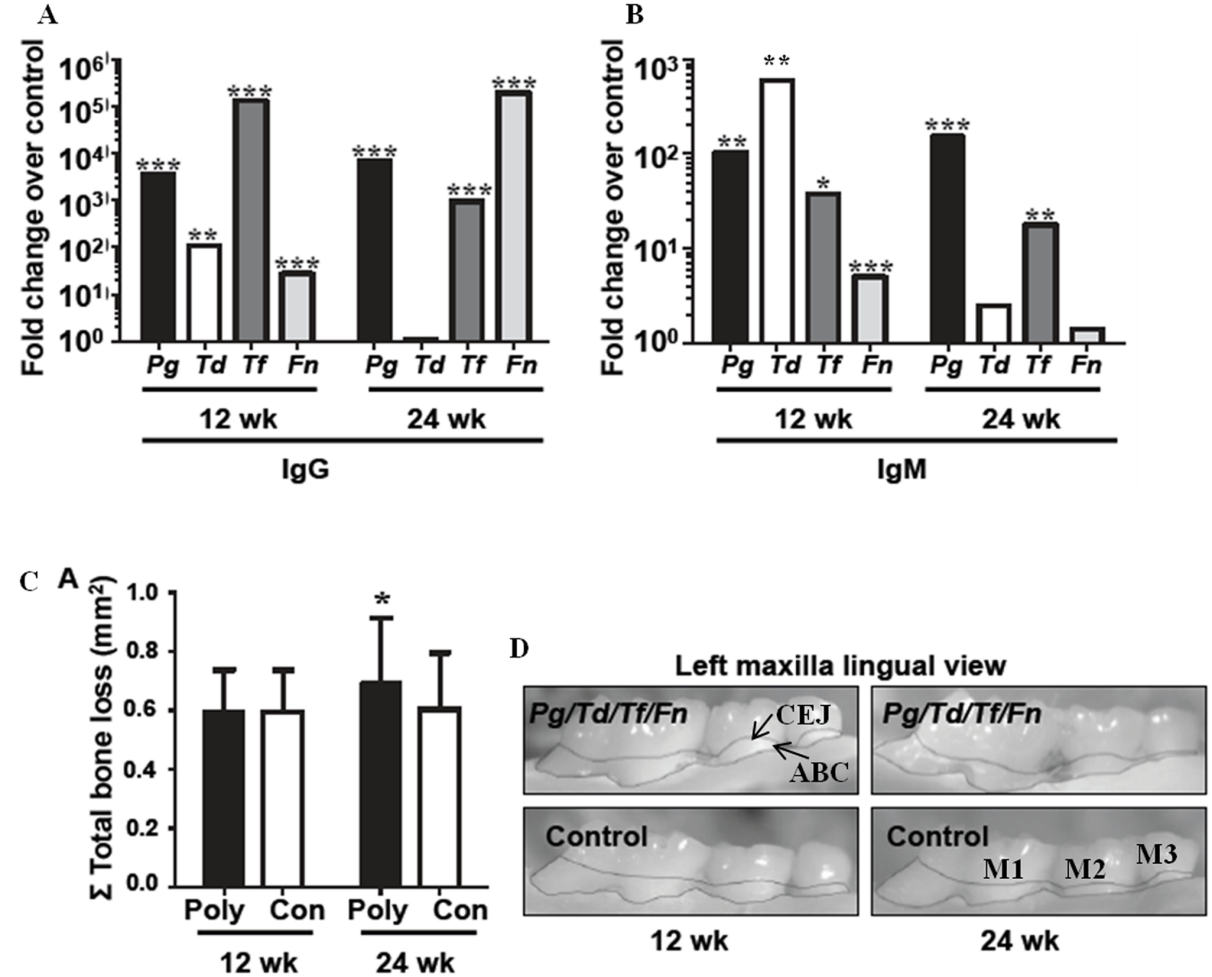
Polybacterial-infection induced bacterial-specific humoral immune response and alveolar bone resorption. (A). Serum IgG antibody response to the four bacteria used in periodontal infection at 12 and 24 weeks. (B). Serum IgM antibody response to the four bacteria used in periodontal infection at 12 and 24 weeks. Representative left maxilla lingual view of polybacterial-infected and sham-infected mice at both 12- and 24 weeks with area of bone resorption outlined from alveolar bone crest to cementoenamel junction. (C). Total alveolar bone resorption is significantly greater than controls at 24 weeks of polybacterial infection. (D). Representative left maxilla lingual view of polybacterial-infected and sham-infected mice at both 12- and 24 weeks with area of bone resorption outlined from alveolar bone crest to cementoenamel junction. Poly—polybacterial infection, Con—sham-infected control, *Pg/Td/Tf/Fn*–polybacterial infection *Pg*–*P*. *gingivalis*, *Td*–*T*. *denticola*, *Tf*–*T*. *forsythia*, *Fn*–*F*. *nucleatum*. * p < 0.05, ** p < 0.01, *** p < 0.001.

To confirm generation of immune response against periodontal bacteria, serum IgG and IgM antibody response to each of the infecting bacteria was determined in infected and control mice at 12 and 24 weeks of infection. Infected mice demonstrated significant serum IgG titers (p < 0.01–0.001) against all 4 bacteria relative to sham-infected controls at 12 weeks of infection, and to *P*. *gingivalis*, *T*. *forsythia* and *F*. *nucleatum* at 24 weeks of infection (p < 0.001) (Fig 1A). The IgM response to *P*. *gingivalis*, *T*. *forsythia*, and *F*. *nucleatum* was significant in 12 week infected mice (p < 0.05–0.001) relative to sham-infected mice, while the 24 week IgM response was significant for *P*. *gingivalis* (p < 0.001) and *T*. *forsythia* (p < 0.01) (Fig 1B).

To confirm induction of PD, we measured the total ABR in polybacterial-infected and sham-infected mice at 12- and 24 weeks. While at 12 weeks of infection the polybacterial-infected mice did not have significant ABR when compared to sham-infected mice (Fig 1C and 1D), by 24 weeks, polybacterial-infected mice had significant ABR (p < 0.05) (Fig 1C and 1D), indicating induction of PD. Additionally, mice with polybacterial infections developed more sites of vertical ABR (intrabony defects), than sham-infected mice at both 12 (13% vs 4% of sites) and 24 weeks (17% vs 5% of sites) of infection (Table 2). Intrabony defects indicated more severe PD in humans, and further substantiate PD induction in our murine chronic periodontal infection model. Together, bacterial colonization in the gingival cavity, IgG and IgM antibody response, and alveolar bone resorption indicate bacterial invasion of gingival epithelium, induction of specific immune response, and progression of PD in the mice.

**Table 2:** Polybacterial infection-induced intrabony defects.

Group	12 Weeks	24 Weeks
	+ sites/total sites	+ sites/total sites
*Pg/Td/Tf/Fn*	28/210 (13%)*	(44/264) 17%
Control	5/114 (4%)	(13/252) 5%

* Percentage of tooth surfaces with periodontal intrabony defects.

### Polybacterial infection induces T cell polarization and inflammatory cytokine production

**Fig 2:**
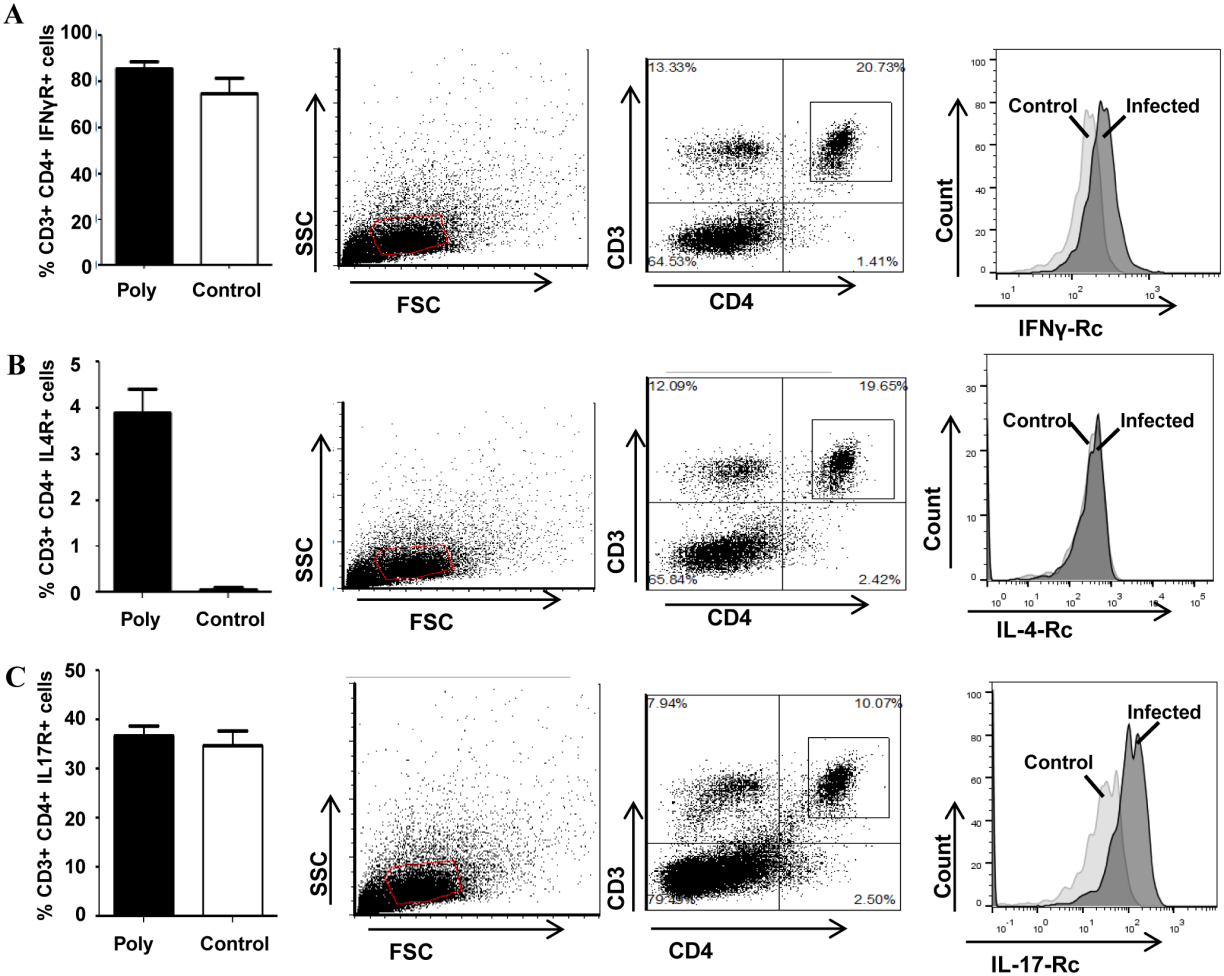
Polybacterial infection elicits a distinct splenic T cell response. Representative images of the gating scheme are shown in panels 2 and 3, with panel 2 showing the lymphocyte gate, panel 3 showing the CD3^+^ CD4^+^ lymphocyte gate, and panel 4 showing the histogram of CD3^+^ CD4^+^ Receptor^+^ lymphocytes. (A). CD3^+^ CD4^+^ IFNγR^+^ cells indicate Th1 response. (B). CD3^+^ CD4^+^ IL4R^+^ cells indicate Th2 response. (C). CD3^+^ CD4^+^ IL17R^+^ cells indicate Th17 response. Poly—polybacterial infection. ** p < 0.01.

In order to evaluate the T cell responses to polybacterial infection, the splenic T cell profile was assessed at 12 weeks of infection by flow cytometry. Specifically, the polarization of the T cell pool to that of a Th1, Th2 or Th17 phenotype was evaluated based on the percentage of T cells expressing IFNγR, IL4R or IL17R, respectively. The percentage of Th1 cells, characterized by expression of IFNγR, was not significantly different between the polybacterial-infected mice and sham-infected mice (Fig 2A). Polybacterial-infected mice had significantly (p < 0.01) elevated levels of IL4R-expressing T cells relative to sham-infected mice (Fig 2B), indicating a strong Th2 response, which aligns with the significant serum IgG and IgM antibody response. IL17R-expressing Th17 cell numbers were not significantly different between polybacterial-infected and sham-infected mice (Fig 2C). Further, we determined serum cytokine alteration by an inflammatory cytokine array to assess how chronic polybacterial infection altered systemic cytokine levels at 12 (n = 6) and 24 weeks (n = 6) of polybacterial infection. At 12 weeks, infected mice demonstrated decreased levels (> 2-fold) of three cytokines, Fas ligand, Interleukin (IL) IL-13, and SDF-1 (Stromal cell-derived factor 1), with increased levels of the chemokine RANTES (Regulated upon activation, Normal T cell expressed and secreted) relative to sham-infected mice (Table 3), indicating an altered inflammatory cytokine response to infection. By 24 weeks of infection, two cytokines involved in attracting and promoting proliferation of innate immune cells, KC (Keratinocyte Chemoattractant) and MCSF (Macrophage colony stimulating factor), were detected at lower levels in infected mice than sham-infected mice (Table 3). Eight cytokines were detected at levels greater than 2-fold higher in infected mice when compared to controls, including the strongly-up-regulated Th2-promoting cytokines IL-3, IL-4, and IL-13, which corresponds to the markedly increased serum antibody response and splenic T cell polarization. Additionally the T cell chemoattractants lymphotactin and RANTES were up-regulated, supporting T cell recruitment in response to the polybacterial infection. Elevated interferon gamma (IFNγ) and IL-1β also indicated infection-enhanced inflammation.

**Table 3:** Polybacterial infection-induced alteration of serum inflammatory markers in ApoE^null^ mice.

12 Weeks		24 Weeks	
Gene Name	Fold Change	Gene Name	Fold Change
Fas ligand	-5.99	KC	-2.24
IL-13	-3.59	MCSF	-2.19
SDF-1	-2.60	GM-CSF	3.15
RANTES	4.13	IFNγ	3.92
		IL-1β	3.58
		IL-3	5.27
		IL-4	15.0
		IL-13	11.0
		Lymphotactin	2.10
		RANTES	2.49

Sera from polybacterial-infected mice (n = 6) and sham-infected mice (n = 6) at both 12 and 24 weeks was analyzed as described in Materials and Methods.

### Evidence of Live Bacteria in Infected Mouse Aortas

**Fig 3:**
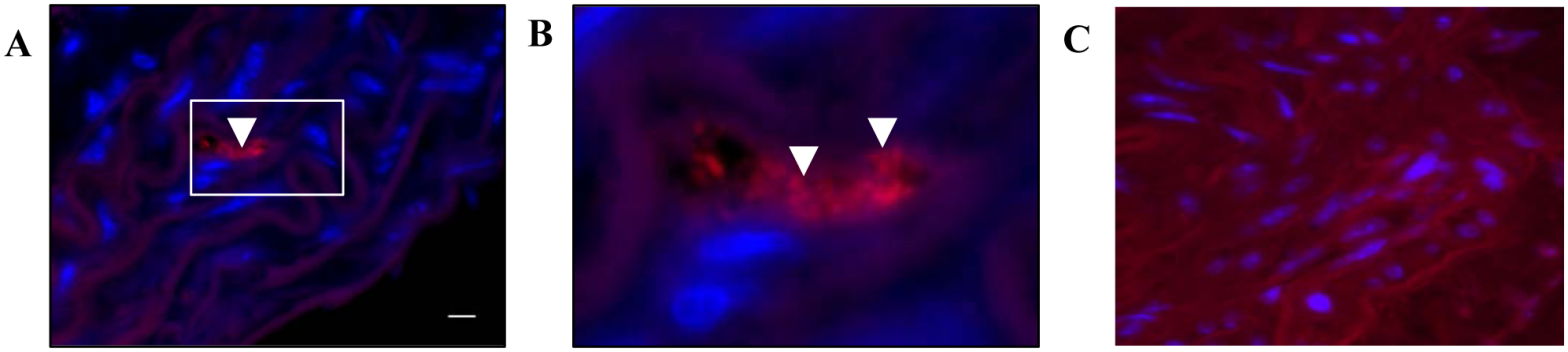
Viable bacteria were recovered from infected mouse heart and aorta. (A). FISH reveals cluster of coccobacilli morphotype of *P*. *gingivalis* in mouse aortic arch at 12 weeks, indicated by white arrow heads. (B). Enlarged inset in D showing clusters of *P*. *gingivalis* coccobacilli morphotype indicated by white arrow heads. (C). No bacteria were detected in sham-infected mouse aortic tissues. Scale bar is 10 micrometers.

Homogenates of heart and aorta were cultured for evidence of viable, invasive bacteria. After 14 days of incubation, colonies were recovered on FAA plates from the aortas (n = 3) and hearts (n = 2) infected mice sacrificed at 24 weeks. Colonies were brown indicating heme-accumulation, with a shiny, mucoid appearance. Colonies were Gram-stained, and revealed Gram-negative species of varying morphotypes (coccobacilli, short bacilli, long slender bacilli). Colonies were tested for the presence of *P*. *gingivalis*, *T*. *denticola*, *T*. *forsythia*, and *F*. *nucleatum* by PCR. Colonies recovered from the aorta of one mouse were positive for both *T*. *denticola* and *F*. *nucleatum* (S1 Table), while a different colony from the aorta and one colony from the heart of the same mouse tested positive for *T*. *denticola* (S1 Table). Colonies recovered from the aorta of a second mouse and the heart of a third mouse were found positive for *F*. *nucleatum* by 16S *rRNA* species specific PCR. Recovering viable oral bacteria from the heart and aorta of mice at sacrifice 14 days after the last oral inoculation demonstrates chronic exposure to and colonization of the bacteria, and their potential to actively invade and persist in vascular tissues. Additionally, FISH was performed on aorta sections of polybacterial-infected and sham-infected mice to detect viable invasive bacteria *in situ* [21]. *P*. *gingivalis* were detected in the aortic wall of one 12 week-infected mouse (Fig 3A and 3B), demonstrating the potential to actively invade and colonize the vasculature. No bacteria were detected in sham-infected mouse aortic tissues (Fig 3C).

### Bacterial Systemic Spread

We determined if bacteria disseminate to systemic organs by collecting heart, aorta, liver, kidneys, and lungs of 3–12 mice in each group at 12 and 24 weeks sacrifice. PCR was performed on the samples to detect genomic DNA of each of the four species used in periodontal infection. *P*. *gingivalis*, *T*. *denticola*, *T*. *forsythia*, and *F*. *nucleatum* genomic DNA was detected in systemic organs by PCR at 12 and 24 weeks (Table 4), and although *T*. *forsythia* was not detected at 12 weeks, it was clearly detected at 24 weeks in heart tissues. Spleens, livers and kidneys of 6 mice in each group at both 12 and 24 weeks were embedded for histology, sectioned, stained with hematoxylin and eosin, and examined for inflammation. No inflammation was detected in examined at either time point (not shown). FISH was performed on sections of the same tissues to detect invasive *P*. *gingivalis*, *T*. *denticola*, *T*. *forsythia* and *F*. *nucleatum*, but no bacterial morphotypes were detected in any of the tissue samples (not shown).

**Table 4:** Distribution of *P*. *gingivalis*, *T*. *denticola*, *T*. *forsythia*, *F*. *nucleatum* genomic DNA in ApoE^null^ mouse tissue by PCR.

		Positive systemic tissue samples					
Weeks	Group	Heart	Aorta	Liver	Spleen	Kidney	Lung
		n = 12	n = 3	n = 12	n = 12	n = 12	n = 12
12	*Pg/Td/Tf/Fn*	7/2/0/0	2/2/0/3	0/0/0/1	3/2/0/0	0/0/0/1	0/0/0/0
	Control	0	0	0	0	0	0
		n = 11	n = 3	n = 11	n = 11	n = 11	n = 11
24	*Pg/Td/Tf/Fn*	7/1/9/0	1/0/1/0	0/2/2/0	0/0/0/0	0/0/1/0	3/1/3/0
	Control	0	0	0	0	0	0

Hematogenous dissemination and invasion of four periodontal pathogens in systemic organs were detected by presence of bacterial genomic DNA by PCR.

### Infection enhances serum atherosclerosis risk factors

**Fig 4:**
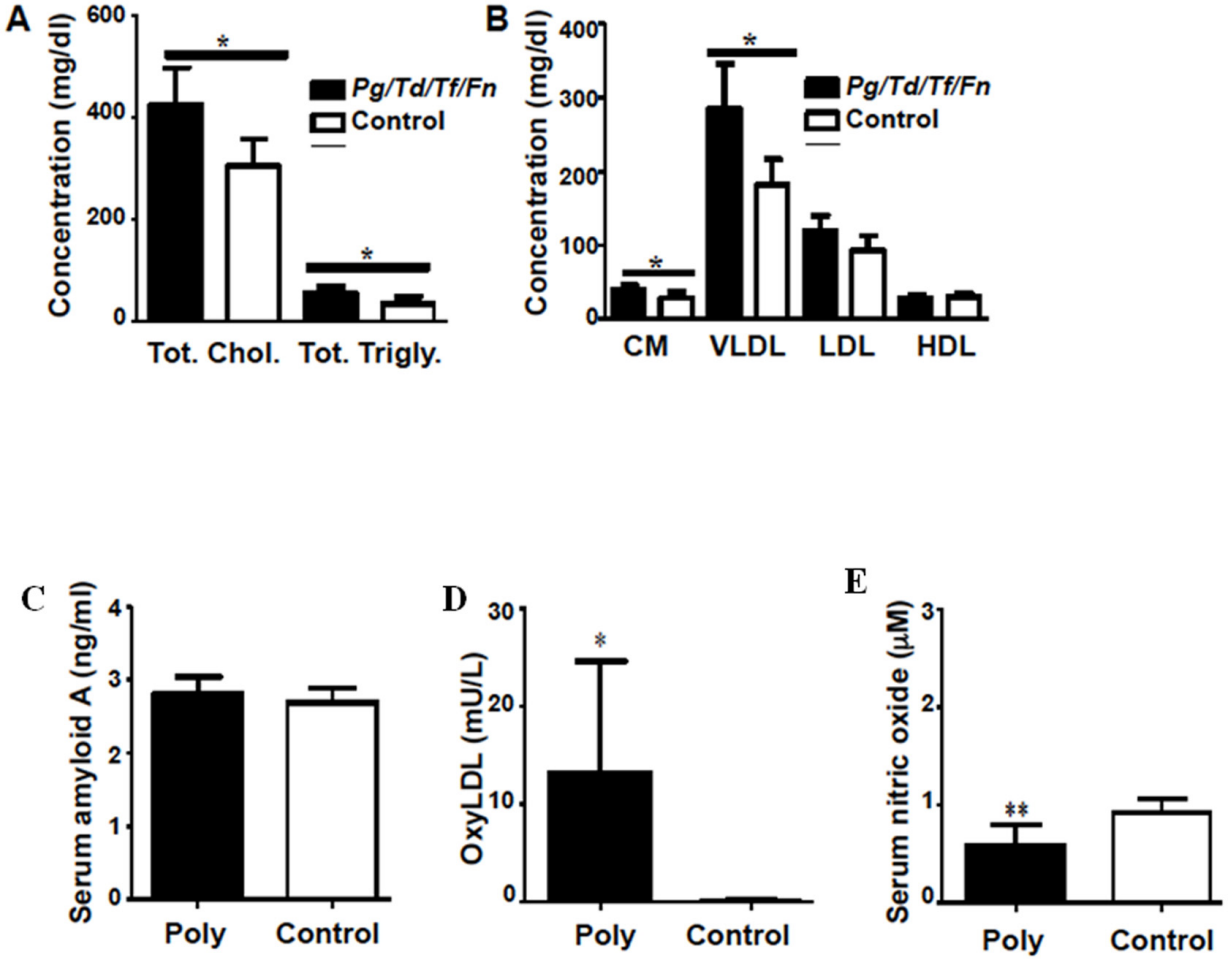
Polybacterial infection enhances atherogenic potential of infected ApoE^-/-^ mice. (A). Total serum cholesterol and total serum triglycerides are significantly elevated in infected mice. (B). Chylomicrons and VLDL are significantly elevated in infected mice. (C). Acute inflammatory marker SAA is not significantly elevated in infected mice. (D). Oxidized LDL is significantly elevated in infected mice. (E). Serum nitric oxide is significantly decreased in infected mice. Chol—cholesterol, Trigly—triglycerides, CM—chylomicrons, VLDL—very low-density lipoprotein, LDL—low density lipoprotein, HDL—high density lipoprotein, Cn—control, Ply—poly infection, *Pg*–*P*. *gingivalis* infection, *Td*–*T*. *denticola infection*, *Tf*–*T*. *forsythia* infection, *Fn*–*F*. *nucleatum* infection, OxyLDL—oxidized LDL. n = 6, * p < 0.05, ** p < 0.01.

The serum lipid profiles, SAA, NO and oxyLDL of twenty-four week-infected (n = 6) and sham-infected (n = 6) mice were assessed to determine if bacterial infection altered known risk factors for atherosclerosis. Serum lipid profile analysis indicated a significant (p < 0.05) increase in total serum cholesterol and triglycerides of infected mice (Fig 4A), indicating they are more prone to develop atherosclerosis. Additionally, chylomicron and LDL particles were significantly (p < 0.05) elevated in infected mice relative to sham-infected mice (Fig 4B), indicating that infection alters the lipid profile to a more pro-atherogenic state. Serum amyloid A is used as a non-specific inflammatory serum risk marker for atherosclerosis in humans; however, the SAA concentration of infected mice was not significantly elevated relative to controls (Fig 4C). Oxidized LDL, a risk factor for atherosclerosis development, was significantly (p < 0.05) elevated in polybacterial-infected mice relative to sham-infected mice (Fig 4D), further supporting that periodontal infection alters lipid transport and metabolism with potential increased risk for atherosclerosis progression. Serum NO was significantly decreased (p < 0.01) in polybacterial-infected mice (Fig 4E), indicating endothelial dysfunction, which is known to be an initiating factor for atherosclerotic plaque progression. These findings indicate that infection may lead to dyslipidemia and endothelial dysfunction, which predisposes the aortic vessel to atherosclerotic plaque formation.

### Polymicrobial infection induces atherosclerotic plaque growth

**Fig 5:**
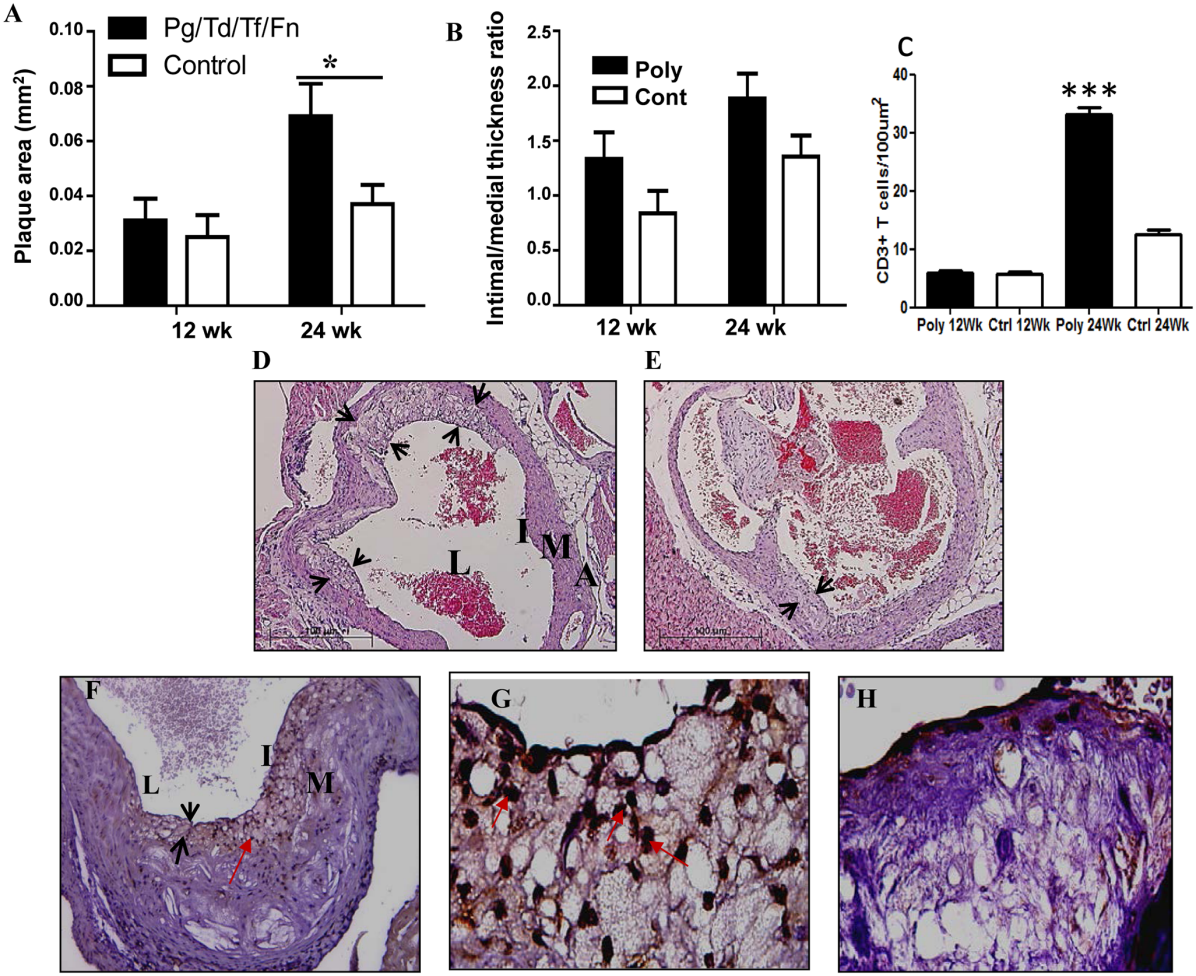
Polybacterial infection significantly increased atherosclerotic plaque growth in the ascending aorta and the aortic root. (A). Total plaque area of infected mice was elevated at both 12- and 24 weeks of infection, but was significant compared to controls at 24 weeks of infection (p < 0.05). (B). Intimal/medial layer thickness ratio of infected mice was increased relative to sham-infected mice at both 12- and 24 weeks of infection, although not significant (p = 0.1599 and 0.1339). (C). CD3^+^ T cell counts were significantly higher in 24 week–infected mice than sham-infected mice. (D). H & E stained aortic section illustrates large plaques in infected mice. (E). H & E stained aortic section showing small plaques in sham-infected mice. (F). Aortic Sections demonstrating numerous CD3^+^ T cells in 24-week polybacterial-infected mouse (20X magnification). (G). Aortic Sections demonstrating numerous CD3^+^ T cells in 24-week polybacterial-infected mouse (200X magnification). (H). Aortic section from sham-infected mice devoid of infiltrating CD3^+^ T cells. Black arrow heads indicate atherosclerotic plaque. Red arrows indicate infiltrated CD3^+^ T cells. L—artery lumen, I—intimal layer, M—medial layer, A—adventitial layer. * p < 0.05.

In order to determine the extent of polymicrobial infection-induced atherosclerotic plaque development, atherosclerotic plaque was measured in sections cut from the aortic root at the level of the valves to the abdominal aorta in infected and sham-infected mice (n = 6, both groups). Minimal plaque was detected in the thoracic and abdominal aorta as has been previously reported, however, plaque was detected in the aortic root and ascending aorta and plaque growth was compared for polymicrobial and non-infected animals at 12 and 24 weeks follow up. Plaque areas as well as intimal and medial thickness were measured, and the intimal thickness / medial thickness ratios were calculated for 12 and 24 week-infected mice. Mice at 12 weeks of infection had larger plaque areas than sham-infected mice, but this did not achieve statistical significance (Fig 5A). In contrast, at 24 weeks infected mice developed significantly increased plaque area (p < 0.05) on comparison to sham-infected mice at 24 weeks as well as when compared to 12 week-infected mice. By 24 weeks of infection, intimal / medial layer thickness ratios also demonstrated a trend toward increased plaque thickness in infected mice when compared to sham-infected mice which was not significant (p = 0.1339) Fig 5B and 5D–5E). Twenty four-weeks-infected mice had significantly (p = 0.0003) increased CD3^+^ T cell counts per high power field (100X HPF) in the intimal layer (Fig 5C and 5F–5G) when compared to the sham-infected mice (Fig 5H).

### Polybacterial Infection-induced Changes in Gene Expression in the Aorta

To determine if oral polybacterial infection altered gene expression in aortic tissues, the RT^2^ Profiler PCR Mouse atherosclerosis array was used to assess transcriptional levels of 84 pre-selected genes associated with atherosclerosis development in aortic tissue at 24 weeks of infection. In infected mice, 6 of the 84 genes analyzed had significantly altered expression (defined as expression fold change over 3-fold) relative to sham-infected controls. A single gene, *Npy*, was up-regulated (Table 5). Neuropeptide Y activates cell proliferation in macrophages, megakaryocytes and VSMCs, and is reported to promote atherosclerosis, as well as contributing to hyperlipidemia [22]. On the other hand, five genes were down-regulated (Table 5), that are involved in lipid transport and metabolism (*ApoA1*, *ApoB*), apoptosis (*Birc3*), and blood clotting (coagulation) (*Fga*, *Fgb*), potentially indicating an attempt to control lipid accumulation and plaque growth induced by infection.

**Table 5:** Polymicrobial infection-induced altered expression of atherosclerosis related genes in ApoE^null^ mouse.

Gene Name	Fold Change
*Apoa1*	-7.64
*Apob*	-6.62
*Birc3*	-10.08
*Fgα*	-10.36
*Fgβ*	-11.06
*Npy*	6.85

Twenty-four week polybacterial-infected and sham-infected aortic tissue samples were processed and analyzed as described in Methods.

## Discussion

We have examined the effects of chronic periodontal polybacterial infection in ApoE^-/-^ mice. We have demonstrated that the immune response to polybacterial infection with 4 bacteria differs significantly from monobacterial infections, and highlights the importance of studying the effects of polybacterial infections. While certain organisms are demonstrated to be strong drivers of atherosclerotic plaque formation based on monoinfection studies [18–19, 23–24], the results we present here demonstrate that the immune response in a polybacterial infection does not resemble that of the previously reported individual (monobacterial) infections, suggesting the existence of synergism during chronic polybacterial infections.

Gene expression of bacterial species is known to change dramatically from monoculture to co-culture with other organisms, including incubation of *P*. *gingivalis* with *T*. *denticola* [25] or with *Filifactor alocis* [26] and *T*. *denticola* with members of the red, orange and yellow complexes [27]. Altered bacterial cell metabolism in co-culture may explain the difference in host response to polybacterial infection, as the proteins expressed and metabolites produced may differ [25, 27], or may be utilized by the other bacterial species [28–29], reducing host exposure to inflammatory agents. Growth of or infection with some combinations of organisms are known to reduce pathogenicity relative to monoculture or monoinfection [30–32], revealing the very limited knowledge available concerning the influence of bacterial-bacterial interactions on bacterial-host interactions.

In polymicrobial and monomicrobial infections, more host genes were down-regulated overall than up-regulated, which may indicate a general response to reduce the inflammation and cell recruitment in vessels in response to bacteremia. Both *T*. *forsythia* and *F*. *nucleatum* infections reduced expression of more genes (8 each) individually than *T*. *denticola* infection (3 genes) or polybacterial infection (1 gene), which corresponds with the reduced plaque detected in *T*. *forsythia* and *F*. *nucleatum* infections [18, 33–34]. Serum cytokine level changes between infection groups also reveal differences that may be important in determining whether atherosclerotic plaque develops. At 12 weeks of infection, each monoinfection exhibited an increase in two unique cytokines [33–34], while polybacterial infection elevated four cytokines. Similar to the differences between monoinfections and polybacterial infection in gene expression analysis, the cytokine response to polybacterial infection shares very little overlap with the other groups, with only MCP-1 up-regulated in *P*. *gingivalis* and *T*. *denticola* monoinfection as well as polybacterial infection [18–19]. Elevated levels of MCP-1 suggest an increase in inflammatory cell recruitment in these infection groups that corresponds with the observed plaque development.

Our data strongly support an early Th2/systemic antibody response to polybacterial infection, demonstrated by significantly elevated serum IgG and IgM at 12 weeks of infection, supported by significantly greater numbers of Th2-promoting CD4^+^ splenic T cells. We see that this is sustained and even enhanced through 24 weeks of infection by the elevated serum cytokine levels. However, it is particularly interesting that the total number of serum cytokines up- or down-regulated at both 12 and 24 weeks of polybacterial infection is less than in *P*. *gingivalis* [19], *T*. *denticola* [18], *T*. *forsythia* [33] or *F*. *nucleatum* [34] monoinfections. This is opposite of the response observed in four monoinfections (*P*. *gingivalis*, *T*. *denticola*, *T*. *forsythia*, or *F*. *nucleatum*), which elicit changes in serum levels of more cytokines at 12 weeks than at 24 weeks, and may indicate that the polybacterial synergism allows the bacteria to repress or escape immune responses.

Enhanced plaque development seen at 24 weeks of infection relative to 12 weeks of infection and demonstrated that elevated inflammation occurred locally, and may be the result of direct bacterial interaction with the aortic vessel wall. We were able to isolate viable *T*. *denticola* and *F*. *nucleatum* from hearts and aortas of mice two weeks after the final periodontal infection, indicating that the bacteria survived long-term in the mice, although we are unable to say whether the bacteria were within the vessel tissue, adhered to vessel walls or carried in blood caught in the organs at isolation. Slow growth of the bacteria in culture may indicate metabolic adaptation to slow growth *in vivo*, a characteristic of biofilm growth, and mechanism of avoiding innate immune detection. Although viable bacteria were detected in the vasculature of only a single mouse, the inclusion is suggestively similar in appearance to the biofilm deposits detected in human coronary vessels by Lanter *et al*., [16], and may indicate that *P*. *gingivalis* is able to establish biofilms in the ApoE^-/-^ mouse aorta, providing an *in vivo* model to study the phenomenon.

Differences in aortic plaque development between this study with infection of 4 bacteria including co-aggregating bacterium *F*. *nucleatum* and our previous study with infection using 3 bacteria [12] are small. The total plaque area with *P*. *gingivalis/T*. *denticola/T*. *forsythia-*infected mice at 18 weeks was close to the total plaque area in 24 week 4 pathogens-infected mice, although the intimal/medial layer thickness ratio of *P*. *gingivalis/T*. *denticola/T*. *forsythia*-infected mice was higher than that of 4 pathogen-infected mice, indicating that the plaques developed in response to infection without *F*. *nucleatum* were thicker.

As *F*. *nucleatum* is considered a “bridging or coaggregating organism” (intermediate colonizer) that promotes attachment of the *P*. *gingivalis/T*. *denticola/T*. *forsythia* (late colonizers) and establishment with in the subgingival biofilm, we expected this polybacterial infection with the 4 pathogens to be more pathogenic than polybacterial infection with 3 pathogens [12]. The reason for this is two-fold. First, we expect *F*. *nucleatum* to coaggregate *P*. *gingivalis/T*. *denticola/T*. *forsythia*, and there by enhance bacterial adherence to the gingival surface, gingival epithelial invasion, and enhance dissemination. Second, *F*. *nucleatum* acts as a pathogen in its own niche and addition of another pathogen could increase the virulence of other pathogens [7]. But in contrast our results in the current study demonstrate that bacterial colonization is enhanced with addition of *F*. *nucleatum* to the red complex bacteria, while periodontal pathogenicity and subsequent atherosclerotic plaque progression were not increased proportionally. These results substantiate our previous findings [34] which have shown inherent atheroprotective effective of *F*. *nucleatum* as a monobacterial infection.

These results reflect an unexpected influence of the addition of another pathogenic species to a known pathogenic bacterial consortium. Although the additional species might help in coaggregation of the bacterial flora as seen *in vivo* but it may also alter the virulence of other members of consortium. This type of unexpected influence may hold true for studies of many polybacterial diseases, including PD, diabetic foot ulcers, and cystic fibrosis, as well as studying normal polybacterial-host/environment interactions such as occur in the human intestines, coral reefs, and acid mine drainage [35]. The only way to discover these effects will be to use polybacterial models to precisely understand the complex interactions within the microbial community and between the microbial community and the host. In summary, this study demonstrates the synergistic effects of polybacterial infections are not the sum of the response to monoinfection with each individual organism, and conclusions drawn from monoinfection studies about the role of any one species in inducing disease may not be accurate regarding the role of that species within a polymicrobial disease. In the future, when drawing conclusion from monoinfection studies about the role of individual microbes in disease pathogenesis, it will be important to keep in mind that pathogenesis of a polymicrobial infection is not sum of infection by its individual species. We would suggest that microbe-microbe interactions appear to alter microbe-host interactions in distinct ways.

## Supporting Information

S1 Table Distribution of ApoE^-/-^ mice heart, aorta samples positive for *T*. *denticola* and *F*. *nucleatum* by PCR. (DOC)
