# Supplementary material for: Polymicrobial Oral Infection with Four Periodontal Bacteria Orchestrates a Distinct Inflammatory Response and Atherosclerosis in ApoEnull Mice
Source: PLoS One. 2015 Nov 30;10(11):e0143291. doi: 10.1371/journal.pone.0143291 (PMC4664240; doi:10.1371/journal.pone.0143291)
Supplement: S1 Table — (DOC) [file pone.0143291.s001.doc]

**S1 Supporting Information**

**S1 Table.** Distribution of ApoE^-/-^ mice heart and aorta samples positive for *T. denticola*, *F. nucleatum* by PCR.

|  | Mouse 1 | | | Mouse 2 | |
| --- | --- | --- | --- | --- | --- |
|  | Aorta | | Heart | Aorta | Heart |
| Colony | 1 | 2 | 1 | 1 | 1 |
| *T. denticola* | + | + | + | - | - |
| *F. nucleatum* | + | - | - | + | + |

(+) - positive PCR band; (-) - negative PCR band.
